# Editorial: Methods in gynecological oncology

**DOI:** 10.3389/fonc.2023.1167088

**Published:** 2023-03-09

**Authors:** Federica Perelli, Alberto Mattei, Giovanni Scambia, Anna Franca Cavaliere

**Affiliations:** ^1^ Azienda USL Toscana Centro, Gynecology and Obstetric Department, Santa Maria Annunziata Hospital, Florence, Italy; ^2^ Department of Science of Woman, Child and Public Health, Fondazione Policlinico Universitario A. Gemelli IRCCS, Università Cattolica del Sacro Cuore, Rome, Italy; ^3^ Division of Gynecology and Obstetrics, Fatebenefratelli Isola Tiberina Gemelli Hospital, Rome, Italy

**Keywords:** gynecological oncology, gynecological cancer, ovarian cancer, endometrial cancer, cervical cancer, vulvar cancer, vaginal cancer

## Introduction

Gynecological oncology deals with the 360-degree management of the woman suffering from a neoplasm of the female genital tract. Its research field is oriented towards an improvement of diagnostic, therapeutic and prognostic techniques aimed at improving the survival and quality of life of women affected by gynecological cancers.

The correct staging and prognostic stratification of cancer patients is the basis for tracing an adequate treatment strategy at the same time radical enough to guarantee the cancer safety and minimally invasive to minimize the negative effects on the quality of life of cancer survivors.

This Research Topic aimed at widening the knowledges on the methods used for scientific advancement in the gynecological oncology field. The issue currently includes 9 papers on the diagnosis, prognostic stratification, treatment strategy of gynecological cancers patients. All contributions to this Research Topic focus on one or more of the research areas highlighted above, evidenced below by reference to the designated areas’ letters.

The gynecological tumors affect female population with different incidences and features. Data from the National Cancer Institute Surveillance, Epidemiology and End Results Program (NCI SEER) reported that the more frequent gynecological malignancy is represented by uterine cancer with 65950 new cases in 2022 and a 5-year relative survival of 81.3%, followed by ovarian cancer with 19880 new cases in 2022 and a 5-year relative survival of 49.7%, cervical cancer with 14100 new cases in 2022 and a 5-year relative survival of 66.7%, vulvar cancer with 6330 new cases in 2022 and a 5-year relative survival of 70.3% (1). Primary vaginal cancer is very rare, showing a prevalence of approximately 1 in 100,000 women (typically of squamous cell histology) (2).

Ovarian tumors show the worst prognosis between gynecological cancers and they can be divided into epithelial, germ cell or sex cord-stromal tumors based on the type of cell from which they originate. Epithelial ovarian cancer (EOC) can be categorized according to their invasiveness and biological features into borderline or malignant.

Gynecological neuroendocrine tumors are rare, there are no clear guidelines for their clinical management and they can originate from cervical, endometrial and ovarian tissue. Ovarian neuroendocrine tumors account for about 2% of all gynecological tumors and they are divided into carcinoid, small cell neuroendocrine tumor (SCNET), and large cell neuroendocrine tumor (LCNET) (3).

## Diagnostic methodology in gynecological oncology

This thematic issue aims to show current advances in diagnosis and screening of gynecologic malignancies.

Xu et al. proposed a radiomics nomogram to distinguish borderline ovarian cancer from malignant epithelial ovarian cancer (EOC). In classifying early-stage type I and type II EOC, the radiomics signature exhibited superior diagnostic performance over the clinical model, based on patient age, menopausal status, CA-125 level, bilaterality, MR-reported pelvic fluid, tumor configuration, and peritoneal involvement. Radiomics has recently emerged as a powerful approach for non-invasively capturing the inter-lesion heterogeneity that can be used to build an objective and accurate decision support systems for cancer patients. The diagnostic efficacy of the nomogram was the same as that of the radiomics model. Xu et al. used imaging features to characterize the properties of adnexal masses with the aim of improving the diagnostic characterization of EOC and providing to tailor specialized treatment plans on different patients. The authors concluded that the radiomics model and combined model had higher benefits than the clinical model to distinguish borderline from EOC.

Concerning diagnostic improvement to better characterize a pelvic mass suspected for malignancy Yang et al. reported a model based on the combination between the marker’s value HE4 and the already proposed ADNEX (Assessment of Different NEoplasias in the adneXa) model proposed by Timmerman et al. in the research conducted by the IOTA (International Ovarian Tumor Analysis) (4–6). The authors concluded that the ADNEX model performs excellently to determine the benignity or malignancy of a ovarian tumor, with higher specificity when combined with HE4. This combination can improve the differential diagnosis ability and the sensitivity of an ovarian borderline tumor and a Stage II–IV OC.

Wang et al. reported a case of superficial myofibroblastoma (SMF) of the lower female genital tract, a relatively rare benign mesenchymal tumor, arose in a 71-year-old Chinese female patient with postmenopausal vaginal bleeding. The authors described a diagnostic path based on gynecological examination and colposcopic evaluation, color Doppler flow imaging, magnetic resonance and the final diagnosis by histopathological analysis. For the first time, colposcopy was used for auxiliary diagnosis and evaluation before surgery for SMF and revealed a lesion covered with normal squamous epithelium with a wide pedicle and a mushroom-like appearance.

## Prognostic stratification in gynecological oncology

This section aims to underline the importance of prognostic classification of gynecological cancer patients in order to tailor them the more appropriate therapeutic and surveillance programme.

Xu et al. with their radiomics nomogram aimed at differentiate borderline ovarian cancer from malignant EOC identified a tool for imaging-based prognostic stratification of patients affected by ovarian malignancies.

Jiang et al. presented a nomogram based on clinical and non-clinical features that affect the prognosis of patients with cervical cancer to develop an accurate prognostic model that correlate with overall survival and cancer-specific survival. The authors constructed nomograms including the following factors: insurance status, grade, histology, chemotherapy, metastasis number, tumor size, regional node examination, LVSI, and radiation.

Pang et al. conducted a retrospective analysis of data from 469 patients affected by gynecologic LCNET to describe prevalence, survival outcomes, and associated factors with this rare neoplasm. Their analysis revealed that American Joint Committee on Cancer stage, lymph node metastasis, and chemotherapy were independent prognostic factors for overall survival and cancer-specific survival in patients with cervical LCNEC. Lymph node metastasis, surgery, and chemotherapy were independent prognostic factors for overall survival and cancer-specific survival in the ovarian group and for overall survival in the endometrial group. Lymph node metastasis and surgery were also independent prognostic factors for cancer-specific survival in the endometrial group.

Li and Cao developed nomograms to predict progression-free survival and overall survival in patients with ovarian clear cell carcinoma after primary treatment. Their analysis included several features studied on 358 Chinese patients. The most predictive nomogram for progression-free survival considered the following variables: thrombosis, the FIGO staging, residual of the tumor and distant metastasis. The most predictive nomogram for overall survival considered the following variables: thrombosis, lymph node metastasis, residual of the tumor, malignant ascites/washing, and platinum resistance.

## Innovative therapeutic strategies in gynecological oncology

This thematic issue aims to propose new treatment strategies for women affected by a neoplasm of the female genital tract.

Pang et al. thanks to their retrospective analysis of data from 469 patients affected by gynecologic LCNET found that surgery alone may help to improve overall survival and cancer-specific survival in patients with early-stage cervical LCNET. In contrast, surgery+chemotherapy and surgery+radiotherapy may help to improve survival in those with early-stage ovarian and endometrial LCNET, respectively. Regardless of subtype, comprehensive treatment involving surgery, chemotherapy, and radiotherapy should be considered to improve prognosis in patients with advanced-stage gynecologic LCNET.

Restaino et al. reported a brilliant and innovative treatment using human amniotic membrane for myocutaneous dehyscence after a radical surgical treatment for vulvar cancer. The authors described the first case of using human amniotic membrane to promote healing of a surgical wound in a patient with gynecological oncology. The implantation of amniotic membranes on surgical wounds appears to be safe, moreover, the psychological impact of the treatment on the patient was acceptable, with an improvement also in terms of pain.

Wang et al. showed the success of the surgical resection treatment of a patient affected by vaginal SMF.

Li et al. compared the survival outcomes among 590 stage IB3 cervical cancer patients who undergo abdominal radical hysterectomy+pelvic lymphadenectomy ± para-aortic lymph node dissection versus radiochemotherapy. The authors concluded that for FIGO 2018 stage IB3 cervical cancer patients, surgery based on abdominal radical hysterectomy and lymphadenectomy resulted in better overall survival and disease-free survival than radiochemotherapy.

Li et al. reported the successful surgical treatment of an ovarian SCNET patients, alive after two years from surgery who underwent laparoscopic total uterine double attachment resection, bilateral ovarian arteriovenous high ligation, abdominal catheterization and postoperative adjuvant chemotherapy.

## Conclusion

In conclusion, this Research Topic provided several investigations focusing on relevant oncological aspects as diagnosis, prognostic stratification and therapeutic strategy of gynecological cancer patients.

Advances in oncological research provide the development of tools for conducting the patient counseling, her postoperative management, and her follow-up to tailor diagnostic, prognostic and therapeutic pathway on each different patient by multidisciplinary approach.

Some other aspects about methods in gynecological oncology should be addressed and deepened in future Research Topic, as the development of minimally invasive surgery techniques, the use of new radio and chemotherapy schemes, the fertility sparing surgical therapeutic pathways in young patients with gynecological cancer.

## Author contributions

All authors listed have made a substantial, direct, and intellectual contribution to the work and approved it for publication.
